# Diagnostic accuracy of the latest-generation digital PET/CT scanner for detection of metastatic lymph nodes in head and neck cancer

**DOI:** 10.3389/fnume.2023.1184448

**Published:** 2023-05-30

**Authors:** Frederick Butt, Lillian Dominguez-Konicki, Noah Tocci, Joseph Paydarfar, Marc Seltzer, David Pastel

**Affiliations:** ^1^ Dartmouth Hitchcock Medical Center, Lebanon, NH, United States; ^2^ Geisel School of Medicine, Dartmouth College, Hanover, NH, United States

**Keywords:** nuclear medicine, head and neck (H&N) cancer, positron emission tomography (PET), radiology, otolaryngology/ENT

## Abstract

**Purpose:**

The aim of this retrospective analysis was to assess the diagnostic accuracy of the latest-generation digital positron emission tomography/computed tomography (PET/CT) scanner in the detection of cervical lymph node metastasis in patients undergoing staging work-up for head and neck cancer.

**Materials and methods:**

A total of 55 consecutive patients with head and neck cancer at our institution who had a PET/CT after installation of the latest-generation PET/CT (Siemens Biograph Vision) who subsequently underwent surgical neck dissection were included. The nodal station location and number of reported PET/CT-positive metastatic lymph nodes were compared to a gold standard of final surgical pathology after neck dissection.

**Results:**

In total, 188 neck levels and 1,373 lymph nodes were resected; 56 neck levels (118 nodes) in 31 (56%) patients contained nodal metastases on surgical pathology. On a nodal level-by-level analysis, the overall sensitivity for the detection of lymph node metastases on the latest-generation PET/CT scanner was 96.4% and the specificity was 86.4%. The sensitivity and specificity for the neck side analysis were 94.0% and 63.7%, and for the individual patient analysis were 100% and 71%, respectively.

**Conclusions:**

In this single-institution study, latest-generation PET/CT had a high sensitivity and moderate to high specificity for detecting cervical node metastasis in head and neck cancer. Compared to data from older PET/CT scanners, the sensitivity of the latest-generation PET/CT was slightly higher, while the specificity was similar or slightly lower. Physicians involved in the management of head and neck cancer should be aware of possible changes in the overall diagnostic accuracy when changing to a latest-generation PET/CT scanner.

## Introduction

Head and neck cancer is an important cause of morbidity and mortality in the United States, with an estimated 65,630 new cases of oral cavity, pharyngeal, and laryngeal cancer in 2020, accounting for 3.6% of all new cancer cases in the USA (1). During the same period, it is estimated that 14,500 deaths from head and neck cancer will have occurred (2). Squamous cell carcinoma (HNSCC) represents the dominant histologic type (>90%) of head and neck cancers, with primary salivary gland cancer and mucosal melanoma comprising the majority of the remaining cases (1, 3). There is a wide range in overall 5-year survival based on the site of the primary tumor with the overall 5-year survival of oral cavity and pharyngeal cancers estimated at 63% (4). The stage at diagnosis further predicts survival in head and neck cancers and is based on the 8th edition of the American Joint Committee on Cancer TNM staging manual published in 2017, which relies heavily on clinical examination and imaging (5). The staging of head and neck cancer involves multiple imaging modalities, including computed tomography (CT), magnetic resonance imaging (MRI), and F-18 fluorodeoxyglucose (FDG) positron emission tomography/computed tomography (PET/CT). MRI and CT with intravenous contrast agents are the preferred imaging modalities for tumor staging (T) because of their intrinsic higher anatomic resolution compared to FDG PET/CT (6). FDG PET/CT is the modality of choice for staging nodal disease (N) with a higher sensitivity than either CT or MRI (7–9). The presence or absence of cervical nodal metastases is the most important factor in the prognosis of HNSCC (10, 11). Lymph node involvement can decrease survival by up to 40% (4, 12). Correct N staging is thus essential to treatment selection and prognostication of a new HNSCC diagnosis.

Much of the existing literature on the accuracy of FDG PET/CT in the detection of cervical nodal metastases in head and neck cancer was obtained on older-generation equipment. Newer silicon photomultiplier-based digital PET/CT scanners have improved spatial resolution and photon detectors (13). With the advent of the newest generation of silicon photomultiplier-based digital PET/CT systems, a concern among otolaryngologists, nuclear medicine physicians, and radiologists is how the improved photon detectors and spatial resolution affect the sensitivity and specificity of nodal metastasis detection in patients with and head and neck cancers. Previous studies were performed using older-generation PET/CT scanners and report that lymph node metastases greater than 12 mm were almost always detectable by FDG PET/CT and nodal metastases less than 6 mm were only detected in half of the cases (14, 15). The improved detection and spatial resolution of the new-generation digital PET/CT system could theoretically lead to increased detection of smaller lymph nodes, both malignant and reactive in etiology. Interpretation of these potentially benign FDG-avid lymph nodes as malignant instead of reactive would lead to increased sensitivity of PET/CT at the expense of the entrusted high specificity. Treatment plans for head and neck cancer are developed based on the known diagnostic performance of PET/CT systems. It is thus important to analyze the sensitivity and specificity of the newest generation of equipment so that head and neck surgeons can make appropriate treatment decisions. The purpose of this study was to report the performance of the latest digital PET/CT scanner in detecting metastatic lymph nodes in patients with head and neck cancer.

## Materials and methods

### Patients

We retrospectively reviewed 55 consecutive patients with head and neck cancer who underwent curative surgery at a single institution between May 2019 and September 2021. We elected to close the study to enrollment 30 months after installation of the new PET/CT scanner. All head and neck surgery patients scanned during that time were included in the study. The study was approved by the institutional review board of Dartmouth-Hitchcock Medical Center. Informed consent was waived due to the retrospective nature of the study. All 55 patients enrolled in the study underwent a PET/CT scan on the latest-generation PET/CT scanner followed by neck dissection or excisional biopsy (41 men, 14 women; mean age=64 years; age range=41–96 years). Surgery was performed with 6 weeks of imaging as part of routine patient care at our institution.

### Image acquisition

All imaging was performed on a digital Biograph Vision PET/CT system (Siemens Healthcare, Erlangen, Germany). Patients fasted for at least 6 h before imaging and the blood glucose level was confirmed to be <200 mg/dl before the intravenous injection of FDG. A weight-based dose of 5.18 MBq/kg (0.14 mCi/kg) was administered 1 h before imaging. Per institution standard protocol, a standard low-dose CT scan was obtained (tube voltage of 120 kV, tube current auto modulation, and spiral pitch factor of 1). This was used for attenuation correction and lesion anatomic characterization. Subsequently, a PET scan was performed. The acquired PET data were reconstructed using the vendor-recommended clinical reconstruction protocol.

### Surgery/pathologic examination

Patients underwent neck dissection and/or excisional biopsy based on the preoperative clinical and radiologic findings. All operations were performed by two head and neck surgeons at a single institution with a combined 50 years of experience. All resected lymph nodes were labeled by the head and neck surgeons based on the standard nodal classification system allowing correlation of pathologic findings with preoperative imaging results (16). The resected lymph nodes were examined with a conventional hematoxylin and eosin stain for metastatic involvement. The pathologic results were provided as a total number of dissected lymph nodes and metastatic lymph nodes by neck nodal level. Metastatic lymph node involvement on a level-by-level, side-by-side, and patient-by-patient basis was retrospectively recorded from the histopathologic reports.

### Image interpretation and analysis

All FDG PET/CT reports were retrospectively reviewed. All PET/CT exams were interpreted by two board-certified nuclear medicine physicians (with 55 years of combined experience) at the time the imaging studies were performed.

The interpretation of a cervical lymph node as benign or malignant was based on the expert opinion of the interpreting radiologist, with primary visual analysis classifying a cervical lymph node as malignant if it had an FDG uptake greater than the blood pool background uptake in the internal jugular veins. Any FDG-avid cervical lymph nodes not described as definitively benign by report were considered positive for metastatic involvement for the purposes of this study, as this reflected clinical practice. Any cervical lymph nodes reported as equivocal in this study were either removed surgically or underwent fine-needle aspiration (FNA).

The PET/CT nodal status results were compared to the gold standard histopathologic reports from the neck dissection surgical specimen. True-positive (TP) cervical lymph nodes were those reported as metastatic by PET/CT and confirmed as metastatic by pathology. Cervical lymph nodes reported as metastatic by PET/CT but without pathologic confirmation were recorded as false positive (FP). True-negative (TN) cervical lymph nodes were those reported normal by PET/CT and pathology. Cervical lymph nodes found to have metastatic involvement on pathology but interpreted as benign on PET/CT were recorded as false negative (FN).

**Figure 1:**
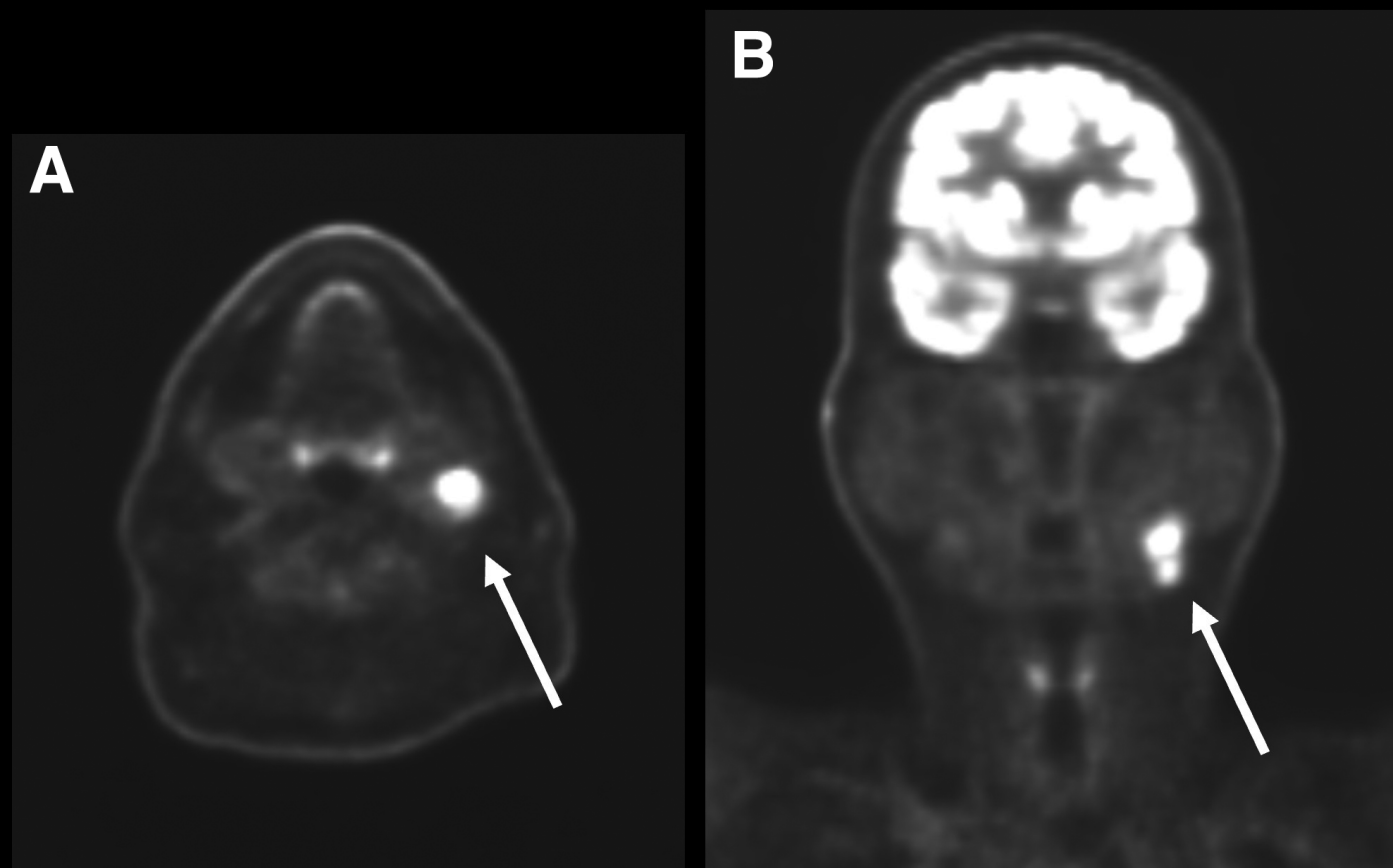
Axial (**A**) and coronal (**B**) PET images of the head and neck in a 66-year-old man with primary left tongue squamous cell carcinoma (not pictured). There are on the left level 2 hypermetabolic lymph nodes (arrows) which were interpreted as nodal metastases by the interpreting radiologist. Surgical pathology confirmed nodal metastatic disease at this level and this patient was classified as a true positive.

For the nodal level statistical analysis, a nodal station level considered positive by imaging and confirmed pathologically was recorded as a TP regardless of the total number of metastatic nodes reported at that level on the pathology report (Figure 1).

**Figure 2:**
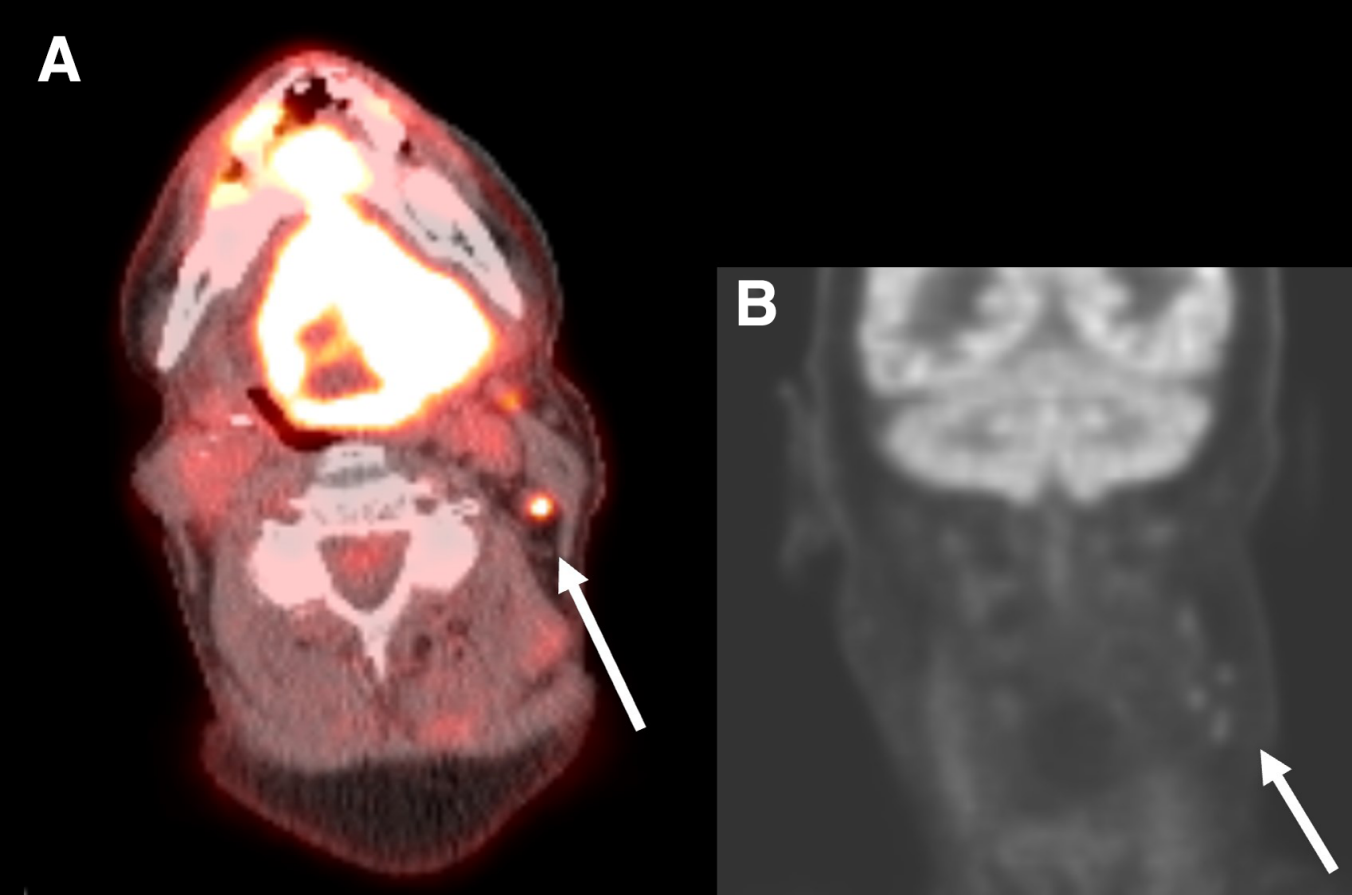
Axial PET/CT fusion of the neck (**A**) and coronal PET image of the head and neck (**B**) in a 67-year-old man with a primary SCC involving the entire tongue. Small hypermetabolic lymph nodes are present on the left at levels 2 and 3 (arrows). These were interpreted as suspicious for malignancy by the interpreting radiologist. On surgical pathology no metastatic lymph nodes were present and this patient was classified as a false positive.

For the neck side statistical analysis, a neck side was considered to be a FP if any ipsilateral cervical lymph nodes at any nodal station were reported as positive on imaging in a patient with negative surgical pathology (Figure 2).

Finally, for patient statistical analysis, a patient was considered to be a FP if any cervical lymph nodes were reported as positive by PET/CT in a patient pathologically negative for nodal disease. A patient with at least one positive cervical lymph node on imaging and confirmed pathologically was recorded as a TP, regardless of the total number of metastatic cervical lymph nodes reported on the pathology report.

This method of patient statistical analysis was chosen to better replicate real-world management decisions. For example, a patient with positive cervical lymph nodes reported at levels 1–3 on PET/CT, but only at levels 2 and 3 on the pathology report, would have a FP recorded by the nodal level analysis but not by the patient-based analysis.

### Statistical analysis

The sensitivity (TP/(TP + FN)), specificity (TN/(TN + FP)), accuracy (TP + TN/(TP + FN + TN + FP)), positive predictive value (TP/(TP + FP)), negative predictive value (TN/(TN + FN)), positive likelihood ratio (sensitivity/(100-specificity)), and negative likelihood ratio ((100-sensitivity)/specificity) were calculated.

## Results

Of the 55 patients included in the study, 11 had bilateral neck dissections and 44 had a unilateral neck dissection. In total, 188 neck levels and 1,373 lymph nodes were resected; 56 neck levels (118 nodes) in 31 (56%) patients contained nodal metastases on surgical pathology. The mean number of lymph nodes removed per patient was 25 (range 2–63), and the median number removed per patient was 24.5 (Table 1).

**Table 1:** Characteristics of patients included in the study.

Variable	Overall
Patients (male/female)	55 (41/14)
Total neck levels dissected (per patient)	188 (3.4)
Total neck sides dissected (bilateral/unilateral)	66 (11/44)
Total lymph nodes dissected (per patient)	1,373 (25.4)

On a nodal level-by level analysis (Table 2), FDG PET/CT prospectively identified 54/56 (96.4%) nodal metastatic levels. One of the two FN lymph nodes had a metastatic deposit measuring 0.2 mm. Both FN lymph nodes abutted an adjacent TP nodal level.

**Table 2:** Diagnostic performance of latest-generation PET/CT scanner for detection of nodal metastases in head and neck cancer patients.

Stratification	TN	FN	FP	TP	Sensitivity (%)	Specificity (%)	Accuracy (%)	PPV (%)	NPV (%)
Nodal level	114	2	18	54	96.4 (87.7–99.6)	86.4 (79.3–91.7)	89.4 (84.1–93.4)	75.0 (66.1–82.2)	98.3 (93.6–99.6)
Neck side	21	2	12	31	94.0 (79.8–99.3)	63.7 (45.1–80.0)	78.8 (70.0–87.9)	72.1 (62.0–80.4)	91.3 (72.8–97.6)
Patient	17	0	7	31	100.0 (88.8–100.0)	70.8 (48.9–87.4)	76.4 (63.0–86.8)	81.6 (70.4–89.2)	100.0

In total, 18 FP nodal levels were identified across 11 patients, yielding a nodal level specificity of 86%. Five patients had a single FP level identified, five patients had multiple FP ipsilateral levels identified, and one patient had FP levels identified on both neck sides.

**Figure 3:**
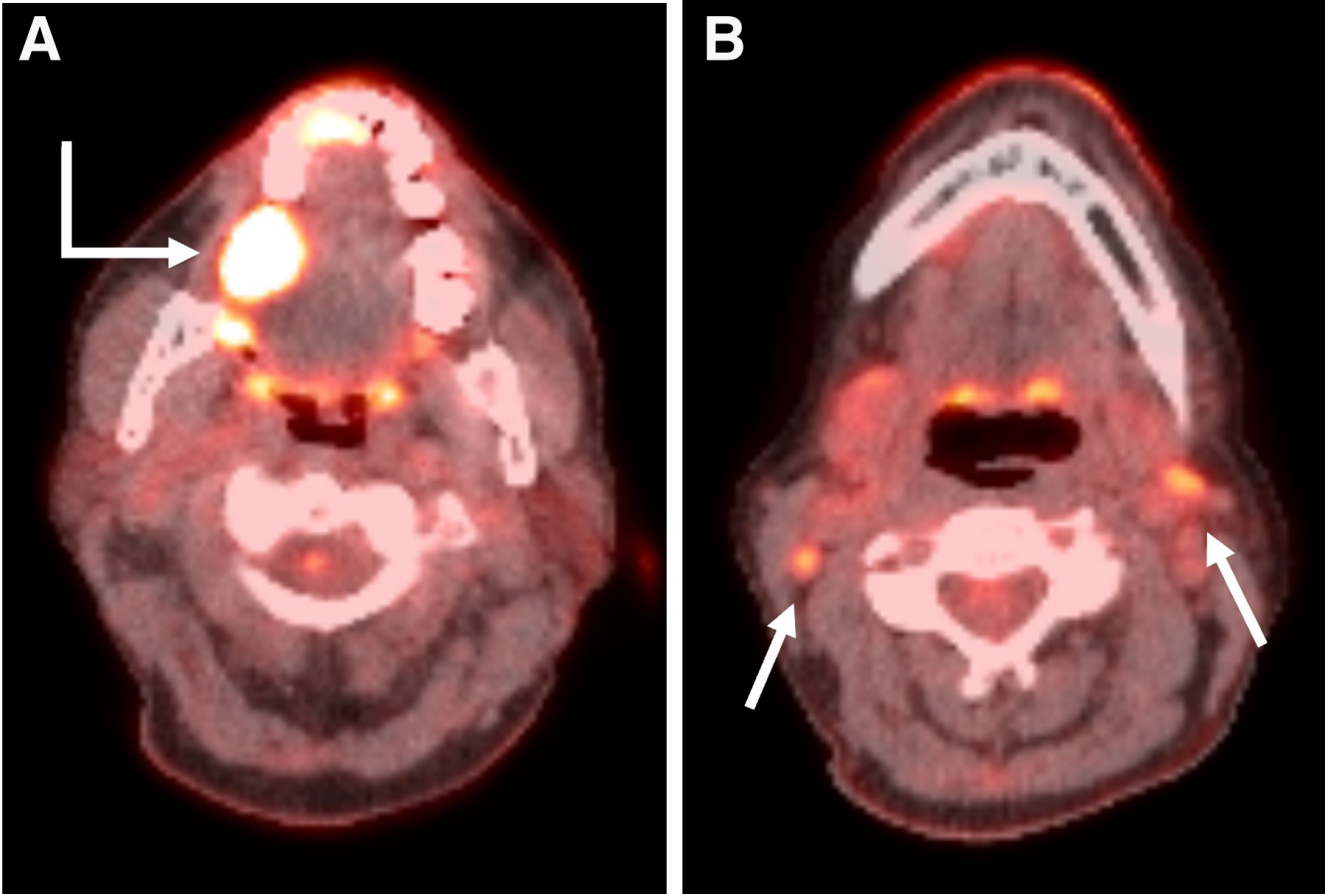
Axial PET/CT fusion images through the oral cavity (A) and neck (B) in a 59-year-old woman with a primary SCC in the right retromolar trigone (Bent arrow). Axial image through the oral cavity demonstrates the primary malignancy. Axial image through the neck demonstrates small bilateral level 2 hypermetabolic lymph nodes (Straight arrows). These were interpreted as suspicious for malignancy by the interpreting radiologist and the patient underwent bilateral neck dissections. No nodal metastases were present on surgical pathology.

FDG PET/CT prospectively identified nodal malignancy in 31/33 (94%) neck side dissections with positive surgical pathology (Table 2B). FDG PET/CT findings were correctly reported as negative for malignancy in 21/33 (64%) neck side dissections. Of the 12 FP neck sides, there were four patients who underwent ipsilateral neck dissection and six patients who underwent bilateral neck dissections. Of the six patients, one had an ipsilateral FP, three had a contralateral FP, and two were confounded by prior malignancy. One patient had bilateral FP neck sides (Figure 3).

**Figure 4:**
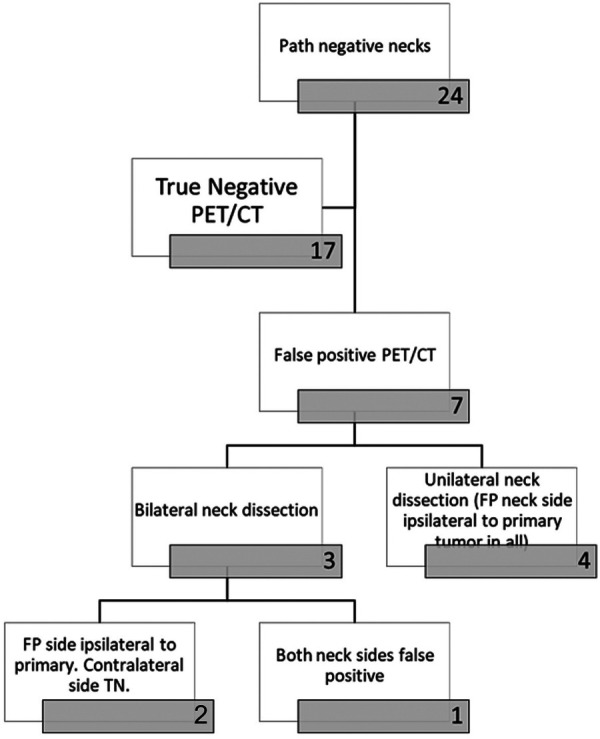
Flow chart demonstrating the breakdown of patients with negative necks on pathology (no dissected lymph nodes positive for malignancy).

All 31 patients with nodal metastatic disease had metastatic lymph nodes preoperatively identified on PET/CT for a sensitivity of 100% (Table 2). PET/CT incorrectly identified the presence of nodal metastatic disease (FP) in 7/24 patients without pathological confirmation of disease, yielding a specificity of 71%. Of these seven patients, three underwent bilateral neck dissections, with two of the three having FP ipsilateral necks but TN contralateral necks. PET/CT incorrectly interpreted both sides as harboring nodal disease in the third patient undergoing bilateral neck dissection (Figure 4).

## Discussion

This is one of the first studies assessing the sensitivity and specificity of the newest-generation digital PET/CT scanners in detecting cervical nodal metastases in head and neck cancer. Many prior studies have analyzed the diagnostic performance of older PET/CT systems. These previous studies have consistently demonstrated a high specificity of PET/CT in detecting cervical nodal metastases, typically in the range of 85%–90% for patient and neck side-based analyses and greater than 95% for a level-based analysis (7–9, 17). The high specificity of PET/CT has been especially useful in reducing FP results and preventing unnecessary neck dissections. The sensitivity of PET/CT for these older systems is lower, with meta-analyses showing a pooled sensitivity of 60%–70% for patient and neck side-based analyses, respectively, and 53% for level-based studies.

Our results support the hypothesis that the improved photon detectors and spatial resolution of the newest PET/CT systems increases the sensitivity of cervical nodal metastasis detection at the cost of lower specificity. The sensitivity is much improved compared to older scanners: 96.4% when analyzed by nodal level and just under that when analyzed by neck side and patient, at 94% and 92.6%, respectively. Of the 188 levels dissected, there were only two levels that were classified as FN. As detailed above, both of these nodes were immediately adjacent to TP levels, suggesting the possibility of a discrepancy in nodal level labeling between the radiology and surgical specimen. One of the two FN nodes had a pathologically detected metastatic nodal deposit of only 0.2 mm, far below the sensitivity of any modern imaging modality.

The specificity of our newest generation PET/CT scanner was lower than described in previous studies. When analyzed by nodal level, neck side, and patient, the specificity was 86.4%, 63.7%, and 71%, respectively. This lower specificity is likely multifactorial. One factor could be that there was a period of adjustment for the radiologists to recognize the increased spatial resolution and sensitivity of the new scanner. Small benign cervical lymph nodes that may have had no perceptible FDG uptake on the older-generation scanners may have some degree of visible FDG uptake on the latest-generation scanner. This could lead to misinterpretation of benign cervical lymph nodes as potentially malignant if reader interpretation thresholds are not adjusted to take into account the higher sensitivity and resolution of the scanner.

Future studies could evaluate the changes in specificity over time after the installation of a latest-generation scanner. We hypothesize that the decreased specificity we observed could in part be a temporal phenomenon, most evident in the first months after installation.

A primary limitation of the study is its retrospective nature. The diagnostic accuracy of cervical lymph nodes as benign or potentially malignant was made by comparing findings from clinical reports to findings at surgical pathology. Although the reported PET/CT data on whether a lymph node was benign or malignant was extracted to best mimic the real-world clinical context of PET/CT interpretation and its impact on clinical decision making, we were not able to obtain additional quantitative data points such as cervical lymph node size, morphology, or maximum SUV values, as these parameters were not consistently documented in the clinical PET/CT reports. In addition, our institutional protocol does not include iodinated intravenous contrast administration, limiting the morphologic evaluation of cervical lymph nodes.

Lastly, in this study, all indeterminate neck cervical lymph nodes were treated aggressively with either FNA or excisional biopsy at time of neck dissection, possibly introducing a bias that could further contribute to our lower specificity.

The newest-generation PET/CT system demonstrates improved sensitivity and decreased specificity relative to older-generation systems. Nearly all of the published data on the diagnostic accuracy of PET/CT in detection of cervical lymph node metastases in head and neck cancer are derived from these older-generation scanners. Thus, all physicians involved in the care of patients with head and neck cancer should be aware of the differences in the diagnostic performance between the newer and older systems and its potential to alter treatment decisions. At present, the literature regarding the diagnostic performance of the newest-generation PET/CT systems remains limited and further studies, such as a large, multicenter study, will be necessary to substantiate the diagnostic accuracy of these scanners in detection of cervical lymph node metastases in patients with head and neck cancer.

## Conclusion

Given the differences in the sensitivity and specificity of malignant cervical lymph node detection on our newest-generation PET/CT system compared to older systems, we believe it is critical that all physicians involved in the care of patients with head and neck cancer, from interpreting radiologists and nuclear medicine physicians to head and neck surgeons, be aware of the equipment used at their institution. The increased sensitivity means there will likely be fewer patients in whom nodal metastases go undetected, but the decreased specificity means that there may be more patients in whom unnecessary neck dissections are performed. Interpreting physicians likely will need to adjust their own thresholds for benign versus malignant lymph nodes based on the increased spatial resolution of the new digital PET/CT scanners. This could affect the treatment planning of the head and neck surgeons and, as such, close communication between all involved parties is prudent. This is especially true in the period immediately after the installation of a new detector as benign versus malignant interpretation thresholds are recalibrated. Further studies are warranted to confirm this altered diagnostic performance relative to older generation scanners. Additional future studies could include quantification of the size of FP lymph nodes relative to those correctly interpreted on PET/CT or a study evaluating diagnostic performance over time after the installation of a new-generation PET/CT system to assess if there are temporal changes of diagnostic performance.

## Data Availability

The raw data supporting the conclusions of this article will be made available by the authors, without undue reservation.
